# Introduction of the Korea BioData Station (K-BDS) for sharing biological data

**DOI:** 10.5808/gi.22073

**Published:** 2023-03-31

**Authors:** Byungwook Lee, Seungwoo Hwang, Pan-Gyu Kim, Gunwhan Ko, Kiwon Jang, Sangok Kim, Jong-Hwan Kim, Jongbum Jeon, Hyerin Kim, Jaeeun Jung, Byoung-Ha Yoon, Iksu Byeon, Insu Jang, Wangho Song, Jinhyuk Choi, Seon-Young Kim

**Affiliations:** Korea Bioinformation Center (KOBIC), Korea Research Institute of Bioscience & Biotechnology, Daejeon 34141, Korea

**Keywords:** biological data, data repository, national R&D

## Abstract

A wave of new technologies has created opportunities for the cost-effective generation of high-throughput profiles of biological systems, foreshadowing a "data-driven science" era. The large variety of data available from biological research is also a rich resource that can be used for innovative endeavors. However, we are facing considerable challenges in big data deposition, integration, and translation due to the complexity of biological data and its production at unprecedented exponential rates. To address these problems, in 2020, the Korean government officially announced a national strategy to collect and manage the biological data produced through national R&D fund allocations and provide the collected data to researchers. To this end, the Korea Bioinformation Center (KOBIC) developed a new biological data repository, the Korea BioData Station (K-BDS), for sharing data from individual researchers and research programs to create a data-driven biological study environment. The K-BDS is dedicated to providing free open access to a suite of featured data resources in support of worldwide activities in both academia and industry.

## Introduction

Biological data refer to information derived from living organisms and their products. They are generated from various research areas, including genomics, proteomics, metabolomics, and microarray-based gene expression profiling. In the not-so-distant past, data generation was a bottleneck; now, the rapid technological advancements and the price reductions of new technologies used for biological data production have led to the exponential growth of biological data, the most significant current trend observed in the life sciences [1]. In this data-rich era, it is no exaggeration to say that biological information plays an essential role in scientific development, and the future of biological research highly depends on the proper utilization and management of data [2].

Biological data possess unique characteristics that make biological data management a particularly challenging problem. First, massive amounts and multiple types of 'big' data, such as sequence data, protein structures, and bioimages, are generated from a variety of biological studies. Thus, they are highly complex when compared to other forms of data. Second, since complex biological phenomena involve many aspects, they cannot be fully explained using a single data type. For this reason, the integrated analysis of different data types has attracted increased research attention. However, there are difficulties due to the challenges of heterogeneous data and the implicitly noisy nature of biological data.

Many biological databases, such as GenBank [3] and Protein Data Bank (PDB) [4], have been established to share these massive and complicated datasets. They also play a crucial role in scientific research by storing, managing, and making the increasing number of datasets accessible. They also offer access to a wide variety of biologically relevant data, including the genomic sequences of an increasingly broad range of organisms. The International Nucleotide Sequence Database Collaboration (INSDC) [5] is a globally-known biological database. This long-standing foundational initiative operates between the National Center for Biotechnology Information (NCBI) [6], the European Molecular Biology Laboratory's European Bioinformatics Institute (EMBL-EBI) [7], and the DNA Data Bank of Japan (DDBJ) [8]. The organization has worked collaboratively to enable access to nucleotide sequence data in standardized formats for the worldwide scientific community for more than 30 years. In addition, the National Genomics Data Center (NGDC) [9] in China provides a suite of database resources mainly focused on genomics data.

These global data centers have been committed to preserving and providing access to complete public-domain nucleotide sequences and associated metadata, enabling discoveries in biomedicine, biodiversity, and biological sciences. However, these data centers signaled the possible limiting of the free-of-charge dissemination of data due to the increasing cost of data management. Moreover, considerable challenges are being faced in big data deposition, integration, and translation due to the complexity of biological data and its production at unprecedented exponential rates.

Korea is actively producing big data due to large-scale government R&D investments in the biological field. Furthermore, the Korean government officially announced a national strategy for sharing the biological data created using national R&D funds. However, due to the lack of a centralized data center to store such a large amount of data, the reality is that individual researchers own most of the data produced in their biological R&D projects. Thus, the data held by individuals will likely be lost after the completion of the project over time.

To address these problems, the Korea Bioinformation Center (KOBIC) developed a new data repository, the Korea BioData Station (K-BDS), for the last two years and launched it this year. The K-BDS aims to archive biological data through the allocation of national R&D funds and offers unrestricted access to all its publicly available data to the worldwide scientific communities. The purpose of this article is to provide an overview of the recent development of the K-BDS, which is publicly available at https://www.kbds.re.kr.

## Implementation

**Fig. 1.**
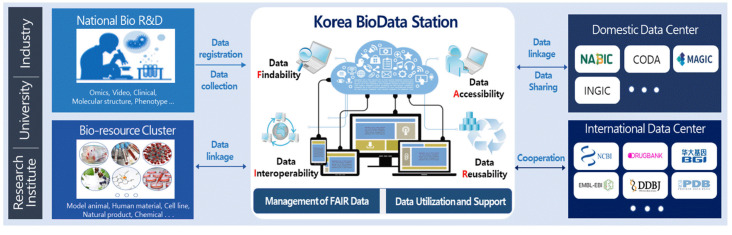
The general architecture of sharing biological data using the Korea BioData Station (K-BDS).

The K-BDS is a public repository that manages the biological research data produced by all Korean government-funded research projects, including medical science, health, agriculture, fishery, environmental biology, and many others (Fig. 1). The main features of the K-BDS are standardized submission forms for each data type, systematic data submission processes and quality control processes, support for the Korea Human Biological Data Bank and the provision of external linkages with the Integrated R&D Information System (IRIS) in Korea.

## Data Organization

All submitted data are roughly organized into two parts, *i.e.*, metadata and files, where the former can be further assorted into BioProject, BioSample, and experiment, and the latter contains raw data. BioProject, bearing an accession number prefixed with PRJK (where the K stands for Korea from now on), provides an overall description of an individual research initiative, including the submitter, basic project description, funding information, and publication(s) if available. BioSample, possessing an accession number prefixed with SAMK, contains a description of the biological material used in the experiments, including organism, sample types, and attributes. The experiment provides a detailed description of treatments for a specific sample, including library methods and experimental intentions. Raw data are a list of the data file(s) related to a specific experiment.

The biological data in K-BDS are classified into five major groups according to data types. The data types are genomics (next-generation sequencing, microarray, nucleotide sequence, polymerase chain reaction primer), proteomics, metabolomics, chemical (bioassay, structure, efficacy test, profiling), and bioimaging (magnetic resonance imaging, positron emission tomography, electroencephalography, intracranial electroencephalography, magnetoencephalography, optical microscopy, electron microscopy, computed tomography, ultrasonography, X-ray, and CryoEM). The K-BDS also has the other data type for any other data except the five groups.

The accession number is a unique identifier assigned to every single submitted record into the K-BDS. The format of the accession number is [alphabetical prefix][series of digits] (Table 1). Each accession number could represent a project, sample, and a hierarchically followed raw data dataset.

## Data Submission Formats

The K-BDS is a repository for all types of data for biological research. Therefore, data submission forms were developed for each of the data types. Depending on the data type, different strategies were adopted to establish a data submission form that could make the inputted information sufficient and valuable. The formats of the data types are divided into three cases. First, for some data types, there is either an official standard file format (for example, DICOM for medical imaging data) or a de facto standard file format (for example, FASTQ for high-throughput genomic sequencing data). Such data types also usually have minimum information guidelines on how to prepare their metadata as well as established data archives specific to them, an example for functional genomics data is the Minimum Information About a Microarray Experiment (MIAME) guideline [10] and the Gene Expression Omnibus (GEO) archive [11]. In such cases, data submission forms in the K-BDS were kept mainly similar to those in the existing standards and archives for international interoperability. Second, some other data types do not have a clear standard. In such cases, experts from the corresponding research field reviewed and assembled existing international or domestic data specifications as well as their perspectives into a novel submission form. Last, other data types are unstructured and *ad hoc*, and their format depends on how the individual researchers organize various information. Such data are usually organized into a tabular spreadsheet form, similar to the supplementary information data that accompanies journal publications. For such unstructured data, the submission forms of the existing generalist repositories, such as BioStudies [12] and Figshare [13], were reviewed and assembled into a novel form for generalist data submission.

In developing the submission forms for all three cases, as mentioned above, for the various data types, experts from diverse areas in the Korean biology community participated in maximizing the submission forms' information content and scrutinizing them. A total of approximately 130 experts participated in the development of the data submission forms from 2020 to 2022. The submission forms are grouped into five major categories of data types (genomics, proteomics, metabolomics, chemicals, and bioimages) and some other categories.

## Data Submission

**Fig. 2.**
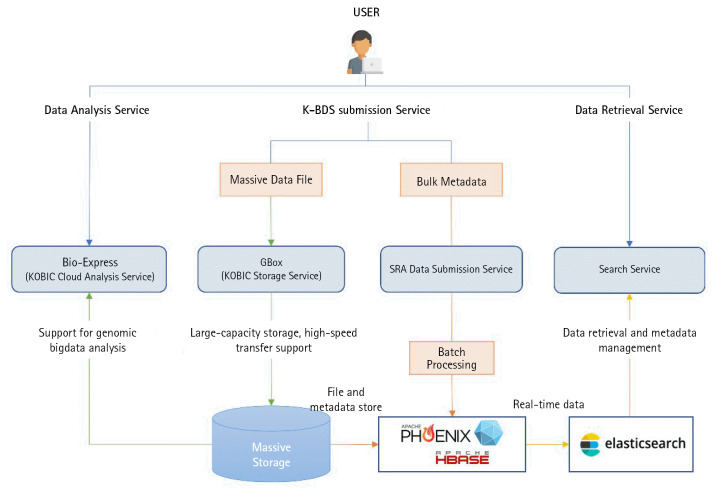
Schematic diagram of the Korea BioData Station (K-BDS), GBox, Bio-Express, and NoSQL database integration platform.

**Fig. 3.**
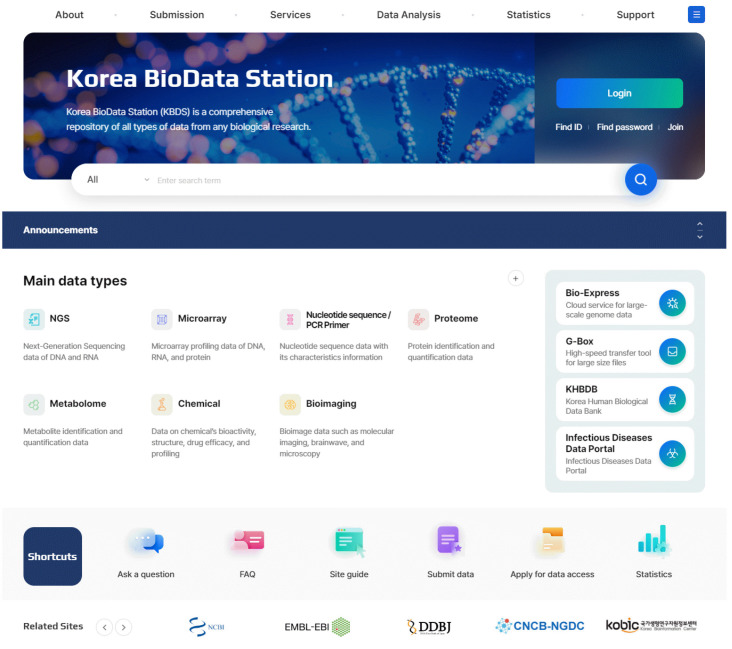
Screenshot of the Korea BioData Station (K-BDS) homepage.

The data submitters upload and submit any biological research data using the K-BDS submission system (Fig. 2). Users must sign up and login into their K-BDS account to submit their data to the K-BDS. Data submission to the K-BDS is comprised of three steps: (1) submit descriptions of biological research projects (BioProject is required), (2) submit a set of descriptions and metadata of biological material (BioSample is required for molecular biological data), and (3) submit a set of biological datasets including raw data and metadata (Fig. 3).

The BioProject is a collection of biological data related to a research effort. A BioProject submission is required for biological data submissions regardless of the data type to be submitted. The BioProject stores information on authors and grants, project information, related publications and patent information if available. A BioProject record provides users with a single place to find links to the diverse data types generated for that project.

BioSample stores the descriptions and metadata about the biological samples used in the studies. BioSample uses a batch submission process and accepts diverse data types, including human, plant, animal, microbe, virus, and metagenome. BioSample examples include a cell line, a primary tissue biopsy, and an individual organism or an environmental isolate. For molecular biology fields such as genomics, proteomics, and metabolomics, a BioSample submission is required before submitting the experiments and raw data. BioSample also provides reciprocal links to BioProject, facilitating sample searches.

The K-BDS has different data submission processes according to the biological data type after inputting data into BioProject and BioSample. The user first selects the data type to be registered, then checks the standard data form to prepare the data to be input, and then inputs the data to the K-BDS registration site. Users can download the user submission manuals for each data type from the K-BDS website. In addition, the K-BDS homepage provides videos for registrants and users so that they can be used more efficiently.

When researchers submit their data, temporal numbers are immediately assigned. These numbers are only used to manage the data and are only revealed to the owners. Then, the accession number is assigned and disclosed to other researchers after completing the quality control process.

As of December 2022, the K-BDS contains 289 biological projects, 45,108 samples, and 1,484,822 data records (Table 2). For NGS genomic data, their accession numbers have been cited in more than 14 articles, including Nature and Blood.

## Uploading Massive Data Quantities to the K-BDS

We developed a fast file transfer tool called GBox, for uploading massive data quantities such as entire genomes and exomes to the K-BDS server from the user's local computer and downloading the selected files to the local computer. The GBox client program can be downloaded from the K-BDS website and installed on the user's computer. The GBox transfer platform provides users with the secure high-speed movement of their data, supporting a wide range of servers, desktops, and Linux operating systems. Using the K-BDS, users can simultaneously upload unlimited numbers of files. GBox has a file transfer speed of approximately ten times that of the standard FTP and HTTP protocols.

## Data Quality Control

The most crucial thing in database development is the quality of the data and its metadata. Data quality indicates a dataset's reliability across key dimensions such as completeness, consistency, and accuracy. Thus, the data quality management system was built to check all the submitted data before issuing an accession number.

The data quality control process in the K-BDS has two steps, the automatic step, where records are automatically checked by software without a manual review, and the expert (or manual) step on selected records. A significant task in the automatic step is to inspect records according to the rules configured in the standard registration form. The submission process does not proceed if the input data are invalid.

For the expert step, domain experts run a comprehensive set of software, search the supporting information from various databases, manually review the results, and interpret the evidence level. The K-BDS has five domain expert groups for genomes, proteomes, metabolomes, chemicals, and bioimages. KOBIC is in charge of genome data, and extramural external expert groups in Korea are designated to perform more professional quality control jobs for the other four data types.

## Data Retrieval

The K-BDS provides user-friendly web interfaces for data queries and browsing. A simple text keyword search allows users to find relevant data. Users can search the data of interest by specifying a given ID of the BioProject, BioSample, or dataset. Moreover, the K-BDS allows users to conduct an advanced search by inputting species names, titles, diseases, and tissue, for example. The K-BDS also allows users to browse all publicly available BioProjects, BioSamples, and datasets.

## K-BDS Implementation

The K-BDS is implemented based on multiple frameworks, the e-Government Standard Framework (eGovFrame; a platform-specific standardized development framework for public sector IT projects in Korea; https://www.egovframe.go.kr/eng/main.do), Java Server Pages (a Java programming framework for constructing dynamic web pages), MyBatis (a persistence framework for the database connection and operation), MySQL (a relational database management system), and Elasticsearch (metadata retrieval for structured/unstructured data). To provide a stable web service, K-BDS is hosted on five servers (CentOS 7), an HTTP server for static content, a tomcat server for dynamic range, a MySQL server for database management, an Elasticsearch server for data retrieval, and an FTP server for data download.

A big-data search engine based on Elasticsearch was built and optimized to enable fast and flexible responses to a given query. The main advantages of the K-BDS retrieval service include: (1) when data are stored in the K-BDS system, they are indexed and fully searchable in near real-time; (2) the support of high-speed retrieval for structured and unstructured data using a big data platform capable of parallel distributed processing; (3) the support of the flexible extension of diverse data types using schemeless NoSQL; (4) the support of diverse programmatic access methods through supplying RESTful APIs; (5) visualization of real-time statistics from access logs using elastic search and Kibana tools.

## Hardware Infrastructure

The hardware design goal of the K-BDS system is to provide large-capacity storage and high-performance computing based on a big data analysis platform for large-scale data. The specifications of the K-BDS infrastructure are as follows: 1,260 CPU cores, 4 NVIDIA A100 80 GB (SXM4) GPUs, 24 TB memory, Mellanox InfiniBand (200 Gb/s), 18.6 PB storage with an additional 18.6 PB backup.

The storage system consists of a DDN ES7990X and a Lustre parallel file system for large-scale data storage and high-speed, low-latency, high-bandwidth data transfer are supported through InfiniBand HDR. The computing servers for extensive data analysis are composed of 1,260 CPU cores, NVIDIA A100 GPUs, 12 TB memory with a Sun Grid engine for distributed processing, and the docker to support various heterogeneous analysis programs. The K-BDS platform uses the PIOLINK PAS-K3200 L4 Switch to perform redundancy and load balancing with the slightest connection algorithm method, WEB (Apache 2.4.53) and WAS (Apache-Tomcat-9.0.68).

MySQL 5.7.26 is used for the database management system. The data backup system consists of primary storage snapshots, backup storage disks and remote backup disks (in the Ochang data centers). To prepare against disasters and disabilities, a disaster recovery system operates in the Ochang data centers. Moreover, K-BDS provides data analysis services with Bio-Express [14], cloud-based large-scale data analysis software, high-speed data transfer with GBox and a flexible search function with a NoSQL database.

Bio-Express is a scalable, cost-effective, and publicly available web service for large-scale genomic data analysis. Bio-Express supports the reliable and highly scalable execution of sequencing analysis workflows in a fully automated manner. The Bio-Express provides a user-friendly interface to all genomic scientists to try to derive accurate results from NGS platform data

## Conclusion

To summarize, the K-BDS is a data repository for archiving raw biological data and providing free access to various database resources supporting research activities. Designed for compatibility, the data structure of the K-BDS adopts international data standards and formats.

The K-BDS has three major advantages. First, the K-BDS is a public repository for the biological research data produced by all Korean government-funded research projects. Therefore, various types of data can be searched on the K-BDS websites. For example, genome, proteome, and image information derived from the same sample can be simultaneously searched and downloaded. Second, the K-BDS provides a web-based user interface for data submission that is simple and easy to use. We also developed a fast file transfer tool, GBox, for uploading massive biological datasets to the K-BDS server from the user's local computer. Lastly, the K-BDS provides a cloud-based analysis function, Bio-Express, for data registered in the K-BDS. Bio-Express enables the data to analyze without downloading the data into the local computer.

The K-BDS is the new data repository that officially started in December 2022. Therefore, the functions and d services of the K-BDS are not the same as those of world-class data centers such as NCBI, EBI, and DDBJ, which have been in operation for more than 30 years. However, due to the active investment of the Korean government and the development of data-based science, the level of KBDS will rise to the level of a world-class data center in a short time.

The ultimate goal of the K-BDS is to establish and promote a centralized archival practice in Korea and support research activities in both academia and industry throughout the world. Ongoing efforts include optimization and automation of the data submission, curation and analysis procedures, an infrastructure upgrade for big data storage and transfer and development of new tools and pipelines to support worldwide biological research.

Sharing data is more useful when others can easily find, access, interpret, and reuse the data. To maximize the benefit of sharing data, the K-BDS will continue to expand and offer a series of data resources and services to benefit a wide range of research initiatives in the life and health sciences.

## Figures and Tables

**Table 1.** The format of the accession numbers of the K-BDS data types

Data types	Accession prefix	Accession format	Example
Temporal number	TEMP	TEMP[0-9]+	TEMP12345
BioProject	PRJKA	PRJKA[0-9]{6}	PRJKA123456
BioSample	SAMK	SAMK[0-9]{8}	SAMK12345678
NGS(Experiment)	KRA	KRA[0-9]{8}	KRA12345678
NGS(Run)	KRR	KRR[0-9]{8}	KRR12345678
Microarray	KAX	KAX[0-9]{8}	KAX12345678
Nucleotide sequences	KNA	KNA[0-9]{10}	KNA1234567899
Primer sequences	KPS	KPS[0-9]{8}	KPS12345678
Proteome(MS)	KPX	KPX[0-9]{8}	KPX12345678
Metabolome(MS,NMR)	KMX	KMX[0-9]{8}	KMX12345678
Chemical compound	KCX	KCX[0-9]{8}	KCX12345678
Bioimage	KVI	KVI[0-9]{8}	KVI12345678
Generalist data	KGD	KGD[0-9]{8}	KGD12345678
The others*	KDT	KDT[0-9]{3}[0-9]{8}	KDT12312345678
Preclinical data	KPC	KPC[0-9]{8}	KPC12345678
Human-driven Biobank	KHB	KHB[0-9]{4}	KHB1234

* The other data type is any other data type except the explicitly described data types in Korea BioData Station (K-BDS).

**Table 2.** The records of the K-BDS database (as of December 2022)

Data type	Records
Genome	1,264,997
Proteome	5,078
Metabolome	7,111
Biological Images	10,342
Chemicals	197,201
The others	93
Total	1,484,822

K-BDS, Korea BioData Station.
